# Adapting the Tools of Our Trade: Shared Decision-Making Aids for Lung Cancer Screening in People With HIV

**DOI:** 10.1016/j.chpulm.2024.100048

**Published:** 2024-03-11

**Authors:** Brett C. Bade, Kathleen M. Akgün

**Affiliations:** a Department of Medicine, Donald and Barbara Zucker School of Medicine at Hofstra/Northwell, New York, NY; b Department of Internal Medicine Section of Pulmonary, Critical Care, and Sleep Medicine, VA Connecticut and Yale University School of Medicine, New Haven, CT; c VA Connecticut Healthcare System, West Haven, CT

Shared decision-making (SDM) for lung cancer screening (LCS) is a difficult task. Before obtaining a low-dose CT scan for LCS, clinical guidelines and the Centers for Medicare and Medicaid Services alike recommend a SDM visit between a patient and their provider that addresses multiple topics, including tobacco smoking, potential benefits and risks (eg, potential procedures, false-positive findings, overdiagnosis), and incorporation of an individual’s preferences and values.^1^^,^^2^ In part due to the complexity of the visit, several studies have shown that information conveyed during the SDM visit is variable (eg, patients’ knowledge about LCS and the recommended follow-up period).^3^^,^^4^ To enhance patient understanding, decision aids are recommended to guide SDM discussions. A well-known example is the University of Michigan’s online tool, which provides an individualized risk calculation for lung cancer using a validated calculator (ShouldIScreen.com).^5^

In many patients, comorbidities must specifically be addressed in the context of SDM for LCS. Why? LCS is intended for people who are at high risk for lung cancer and the likelihood of benefit outweighs the risk of harm. Several comorbidities have been recognized that concurrently (1) increase an individual’s risk of developing lung cancer and (2) have potential increased risk of complications from diagnostic evaluation or treatment. Examples include individuals living with COPD and idiopathic pulmonary fibrosis, but also those aging with HIV infection.^6^ Furthermore, people with advanced, life-limiting comorbidities may face a higher risk of dying from these medical conditions, rather than from a lung cancer identified during screening. Both situations illustrate how screening may represent an unfavorable risk-benefit ratio for selected patients, and providers must estimate how each condition will impact an individual’s course. The challenge of comorbidities in LCS risk-benefit quantification is reflected in professional guidelines. The 2021 American College of Chest Physicians guidelines^7^ do not recommend LCS in several groups where risk likely exceeds benefit due to patients living with life-limiting conditions (eg, advanced liver disease, advanced congestive heart failure, COPD with hypoxemia and hypoventilation). Recognizing that little data are available to guide SDM visits in patients with multiple or severe comorbidities, an ATS research statement^8^ proposed a risk-benefit schema that encompasses risk of developing lung cancer and risk of diagnostic- and treatment-related complications. The schema highlights that patients at the highest risk for lung cancer may be the least able to tolerate diagnostic procedures and treatment. Thus, although SDM visits are necessary for LCS acquisition and decision-making, we have been practicing in a clinical environment with the least guidance for patients most in need of thorough SDM discussions.

With this backdrop, disease-specific decision aids have been proposed to support people facing complex LCS SDM. In this issue of *CHEST Pulmonary*, Brown et al^9^ introduce a SDM tool for people with HIV (PWH). With improved antiretroviral therapies, lung cancer has become the leading cause of cancer death in PWH,^10^ underscoring the importance of LCS in PWH. PWH struggle with higher rates of tobacco smoking, carry increased risk of lung cancer independent of tobacco smoking, and develop lung cancer at younger ages.^11^ These factors argue for tailored approaches for LCS in PWH, with contextualized decision-making that addresses HIV-related risk and overall comorbidity burden. Indeed, the same investigators recently identified additional LCS barriers potentially experienced by PWH (eg, multimorbidity, economic instability, mental health concerns, substance use).^12^ Tailored SDM aids for PWH may be needed to address and overcome disease-specific barriers.

In the current study, Brown et al^9^ used qualitative methods to adapt an SDM tool for PWH who are eligible for LCS. Semistructured focus group interviews were conducted among LCS-eligible PWH (n = 43) and separate groups of providers (n = 10), prioritizing SDM and decision aid elements. Framework-guided thematic analysis mapped themes onto the Health Equity Implementation framework.^9^ Among PWH, themes favored decision aid development, including tailoring LCS SDM to PWH, preference for clear and unambiguous LCS recommendations, and positive messaging that emphasizes survival. Providers prioritized personalized LCS risk assessments and point-of-care tools. Overall, there were mixed opinions as to whether HIV-related health effects should be incorporated into the discussion (vs focusing on lung cancer-specific risks).

A notable strength of this study is incorporation of both patient and provider perspectives. Inclusion of feedback from both sides of the discussion will likely optimize the delivery of information and explore and support the preferences of patients. As importantly, this study introduces a distinct aspect of LCS discussions and decision-making tools: adaptability. Without messaging tailored to the patient sitting before us, providers are likely to convey brief (and potentially superficial) recommendations during LCS SDM visits. Also, using the same decision tools for each patient, rigidity may impede individualized discussions. Recognizing the diversity of patients undergoing LCS, shouldn’t we expect similar themes but widely variable priorities of each individual during SDM, reflecting diverse lived experiences? In the current study, differing opinions on the inclusion of HIV-specific risks in SDM illustrate how perspectives vary depending on one’s life experience and position in the examination room. This simple finding highlights the needs for LCS decision-making tools that estimate an individual’s risk of cancer and incorporate several topics that may (or may not) be relevant to an individual patient.

Thus, this study is an important contribution to advancing personalized LCS SDM discussions for PWH. Although this study focused on LCS in PWH with a new and evolving tool to address disease-specific issues, it also offers a roadmap for adapting these SDM tools in other clinical subgroups. If successful, future tools may be developed and implemented in patients with other complex decision-making (eg, idiopathic pulmonary fibrosis, pulmonary hypertension). More broadly, it seems likely that patients prefer LCS SDM tools that facilitate adaptability, allowing information and conversation to be tailored to their needs and questions. In the absence of such adaptable and individualized SDM tools, providers should consider how current LCS discussions can be adapted to an individual person’s needs.

## Financial/Nonfinancial Disclosures

The authors have reported to *CHEST Pulmonary* the following: B. B. reports unrelated investigator-initiated research support from the American Cancer Society and VA Central Office and is the site principal investigator for clinical trials with Biodesix, Delfi Diagnostics, and Nucleix. None declared (K. M. A.).
